# Supplementary material for: Mechanically Tunable, Compostable, Healable and Scalable Engineered Living Materials
Source: Nat Commun. 2024 Nov 12;15:9179. doi: 10.1038/s41467-024-53052-4 (PMC11557937; doi:10.1038/s41467-024-53052-4)
Supplement: Supplementary file 1 — Supplementary Information [file 41467_2024_53052_MOESM1_ESM.pdf]

Supplementary Information

Mechanically Tunable, Compostable, Healable and Scalable Engineered Living Materials

Avinash Manjula-Basavanna[1,2,3*], Anna M. Duraj-Thatte[2], Neel S. Joshi[1*]

[1] Department of Chemistry and Chemical Biology, Northeastern University, Boston, Massachusetts, United States

[2] Department of Biological Systems Engineering, Virginia Polytechnic Institute and State University, Blacksburg, Virginia, United States

[3] Department of Bioengineering, Northeastern University, Boston, Massachusetts, United States

*Corresponding author

mbavinash@northeastern.edu
ne.joshi@northeastern.edu

Supplementary Table 1. Genes and their DNA sequence.

Gene	DNA Sequence
CsgA	GGTGTTGTTCCTCAGTACGGCGGCGGCGGTAACCACGGTGGTGGCGGTAATAA<br>TAGCGGCCCAAATTCTGAGCTGAACATTTACCAGTACGGTGGCGGTAACTCTGC<br>ACTTGCTCTGCAAACTGATGCCCGTAACTCTGACTTGACTATTACCCAGCATGGC<br>GGCGGTAATGGTGCAGATGTTGGTCAGGGCTCAGATGACAGCTCAATCGATCTG<br>ACCCAACGTGGCTTCGGTAACAGCGCTACTCTTGATCAGTGGAACGGCAAAAAT<br>TCTGAAATGACGGTTAAACAGTTCGGTGGTGGCAACGGTGCTGCAGTTGACCAG<br>ACTGCATCTAACTCCTCCGTCAACGTGACTCAGGTTGGCTTTGGTAACAACGCG<br>ACCGCTCATCAGTAC
Linker	GGTGGATCTGGTAGCAGCGGCTCTGGTGGTTCTGGGGGCGGAAGTGGCTCCTC<br>TGGGAGCGGGGGGTCGGGTGGTGGCTCGGGTTCATCTGGTAGTGGCGGTTCG<br>GGT
SpyTag	CGCGGCGTGCCGCATATTGTGATGGTGGATGCGTATAAACGCTATAAA
SpyCatcher	GTGACCACCCTGAGCGGCCTGAGCGGCGAACAGGGCCCGAGCGGCGATATGA<br>CCACCGAAGAAGATAGCGCGACCCATATTAAATTTAGCAAACGCGATGAAGATG<br>GCCGCGAACTGGCGGGCGCGACCATGGAACTGCGCGATAGCAGCGGCAAAAC<br>CATTAGCACCTGGATTAGCGATGGCCATGTGAAAGATTTTTATCTGTATCCGGGC<br>AAATATACCTTTGTGGAAACCGCGGCGCCGGATGGCTATGAAGTGGCGACCCC<br>GATTGAATTTACCGTGAACGAAGATGGCCAGGTGACCGTGGATGGCGAAGCGA<br>CCGAAGGCGATGCGCATACC
Spacer	ATGAAAGTGCTGATTCTGGCGTGCCTGGTGGCGCTGGCGCTGGCGCGCGAAAC<br>CATTGAAAGCCTGAGCAGCAGCGAAGAAAGCATTACCGAATATAAACAGAAAGT<br>GGAAAAAGTGAAACATGAAGATCAGCAGCAGGGCGAAGATGAACATCAGGATAA<br>AATTTATCCGAGCTTTCAGCCGCAGCCGCTGATTTATCCGTTTGTGGAACCGATT<br>CCGTATGGCTTTCTGCCGCAGAACATTCTGCCGCTGGCGCAGCCGGCGGTGGT<br>GCTGCCGGTGCCGCAGCCGGAAATTATGGAAGTGCCGAAAGCGAAAGATACCG<br>TGTATACCAAAGGCCGCGTGATGCCGGTGCTGAAAAGCCCGACCATTCCGTTTT<br>TTGATCCGCAGATTCCGAAACTGACCGATCTGGAAAACCTGCATCTGCCGCTGC<br>CGCTGCTGCAGCCGCTGATGCAGCAGGTGCCGCAGCCGATTCCGCAGACCCTG<br>GCGCTGCCGCCGCAGCCGCTGTGGAGCGTGCCGCAGCCGAAAGTGCTGCCGA<br>TTCCGCAGCAGGTGGTGCCGTATCCGCAGCGCGCGGTGCCGGTGCAGGCGCT<br>GCTGCTGAACCAGGAACTGCTGCTGAACCCGACCCATCAGATTTATCCGGTGAC<br>CCAGCCGCTGGCGCCGGTGCATAACCCGATTAGCGTG

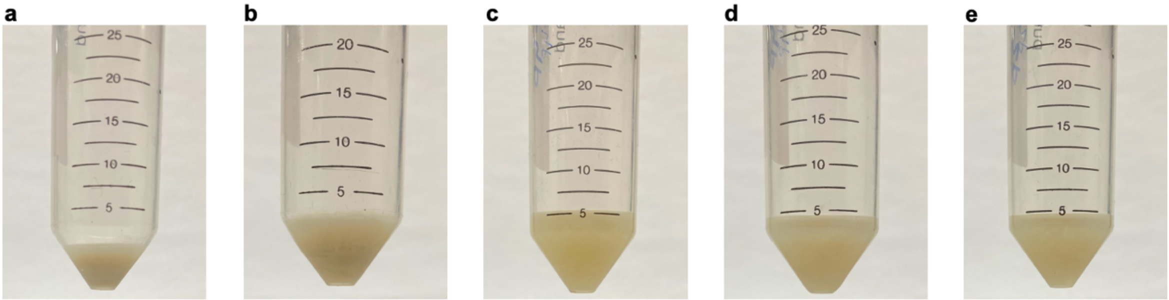

Supplementary Figure 1. Gelator treated curli biomass. Photographs of curli biomass obtained from **a** 1%, **b** 2%, **c** 3%, **d** 4% and **e** 5% gelator (sodium dodecyl sulfate).

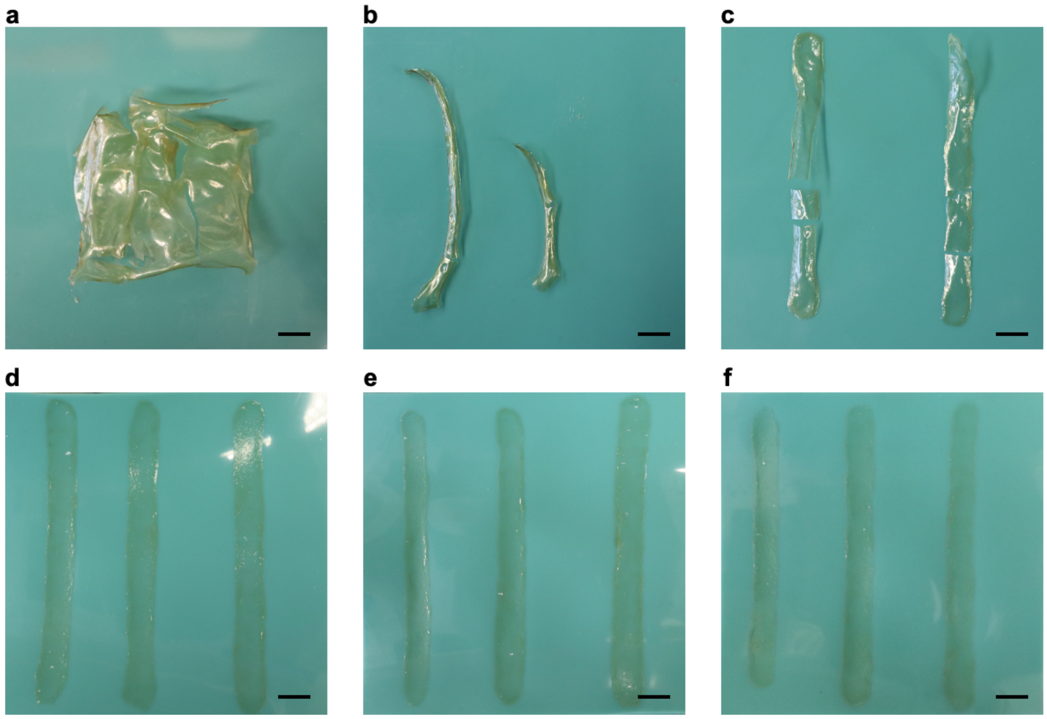

Supplementary Figure 2. Gelator treated MECHS. Photographs of MECHS obtained from **a** 0%, **b** 1%, **c** 2%, **d** 3%, **e** 4% and **f** 5% gelator (sodium dodecyl sulfate). Scale bar 1 cm.

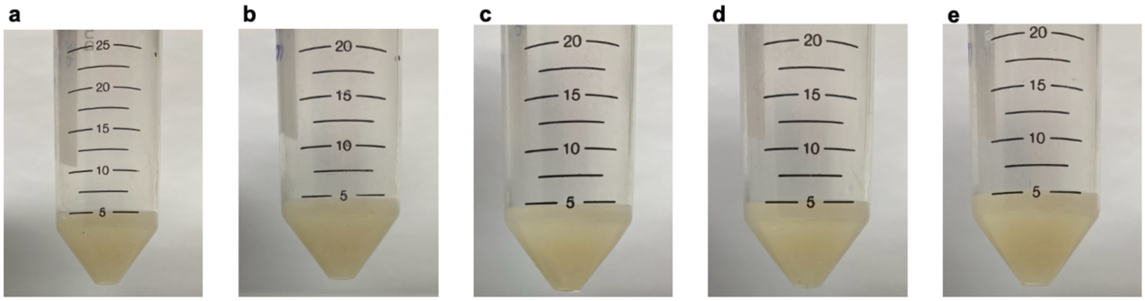

Supplementary Figure 3. Plasticizer treated curli biomass. Photographs of curli biomass obtained from **a** 1%, **b** 2%, **c** 3%, **d** 4% and **e** 5% plasticizer (glycerol).

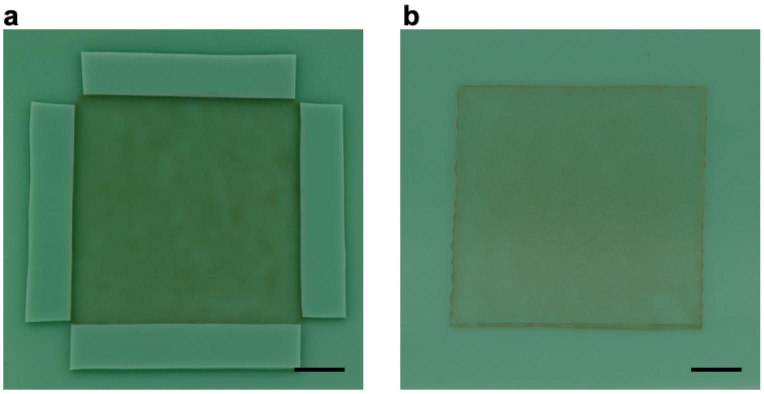

Supplementary Figure 4. Plasticizer treated MECHS. a and **b** Photographs of MECHS film obtained by treating with 3% plasticizer (glycerol) and casting on a silicone mold. Scale bar 1 cm.

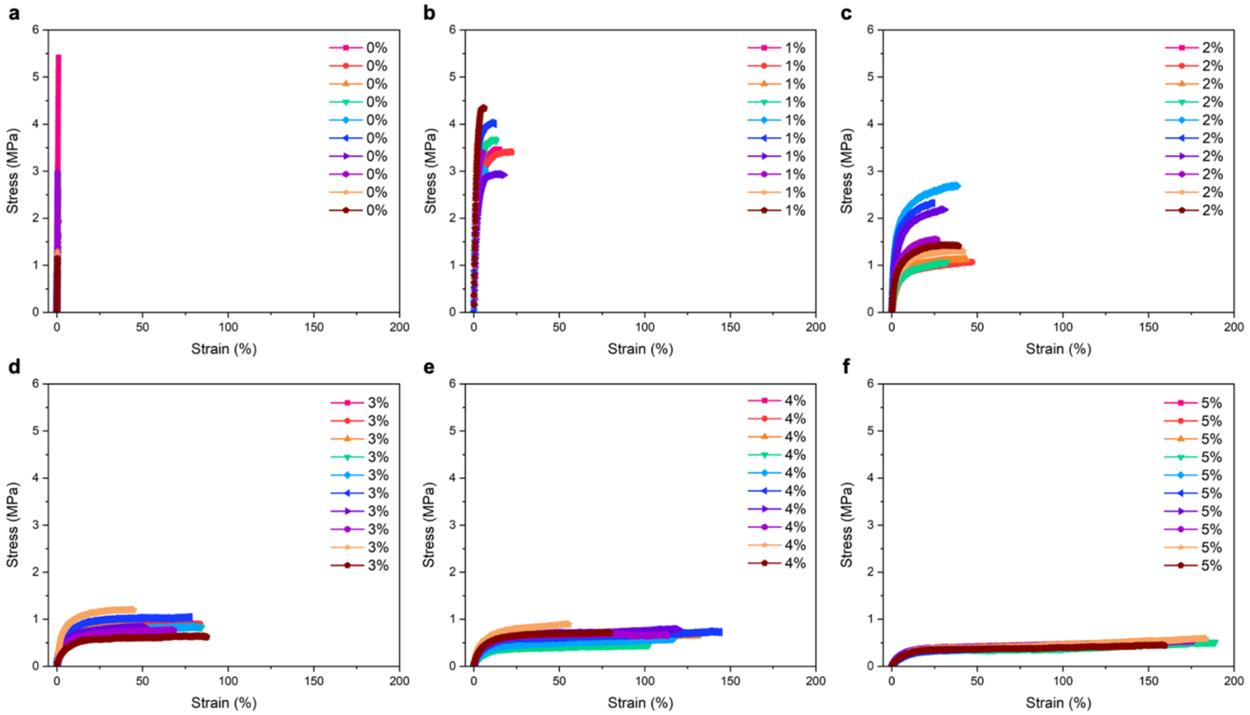

Supplementary Figure 5. Tensile tests of MECHS. Stress strain curves of MECHS films obtained from **a** 0%, **b** 1%, **c** 2%, **d** 3%, **e** 4% and **f** 5% plasticizer (glycerol).

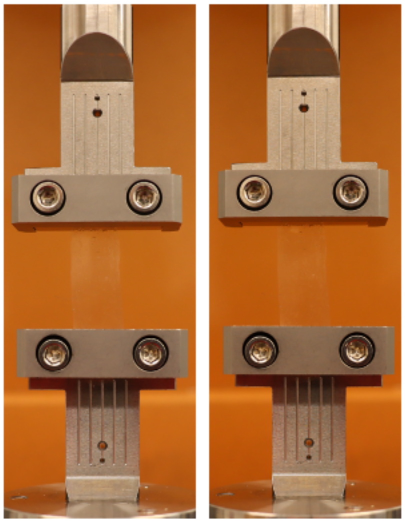

Supplementary Figure 6. Tensile test of MECHS. Representative photographs show the tensile test of MECHS film with the lateral dimension of 1 cm by 4 cm obtained from 3% of gelator. Left image: initial. Right image: before break

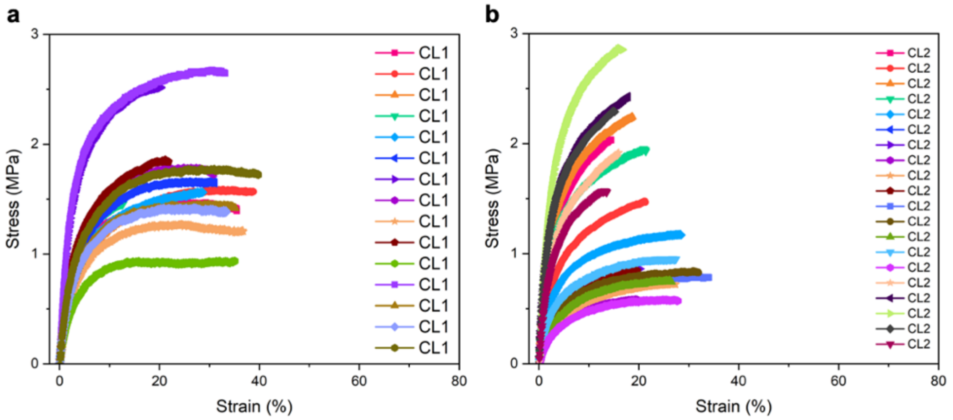

Supplementary Figure 7. Tensile tests of MECHS. Stress strain curves of MECHS obtained from **a** CL1 and **b** CL2.

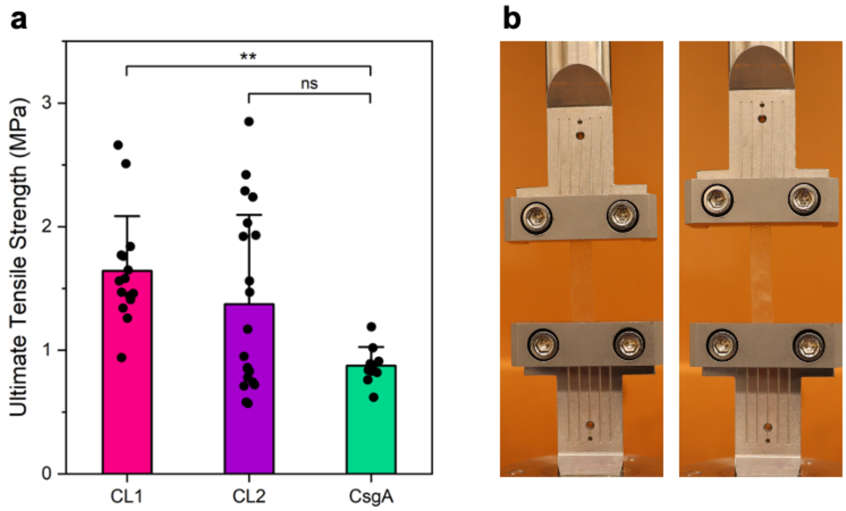

Supplementary Figure 8. Tensile tests of MECHS. a Ultimate tensile strength of CsgA, CL1 and CL2 with 3% plasticizer. Data represented as mean ± standard deviation. Biological replicates *n* = 10 for CsgA, *n* = 15 for CL1 and *n* = 20 for CL2. **p = 0.0042, ^{ns}p = 0.0637. One-way ANOVA followed by Tukey's multiple comparisons test. **b** Representative photographs show the tensile test of CL2 film with the lateral dimension of 0.5 cm by 4 cm. Left image: initial. Right image: before break.

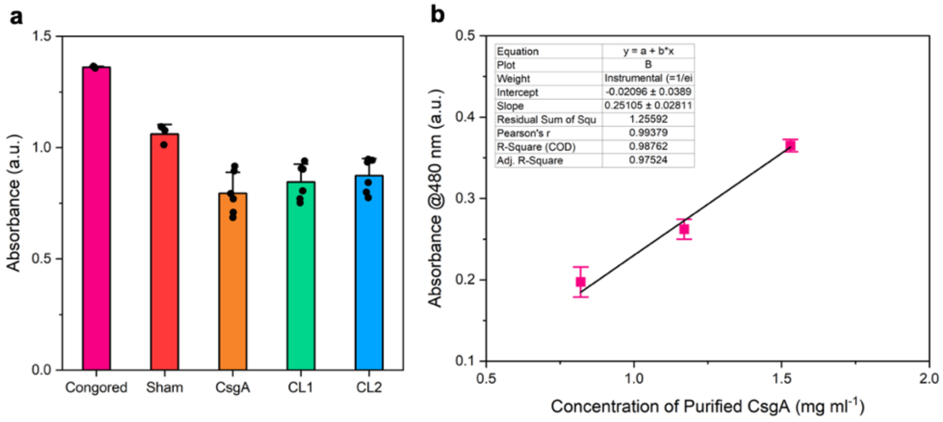

Supplementary Figure 9. Congo Red assay. a Congo Red absorbance at 480 nm for various samples. **b** Congo Red standard curve for purified CsgA (wet weight, CsgA-His). Biological replicates *n* = 3 for Sham and *n* = 6 for CsgA, CL1 and CL2. The net Congo Red absorbance of curli in CsgA, CL1 and CL2 were determined by subtracting the absorbance values of cell pellet having a sham plasmid (without curli operon), to account for the non-specific binding to other biomolecules. Data represented as mean ± standard deviation.

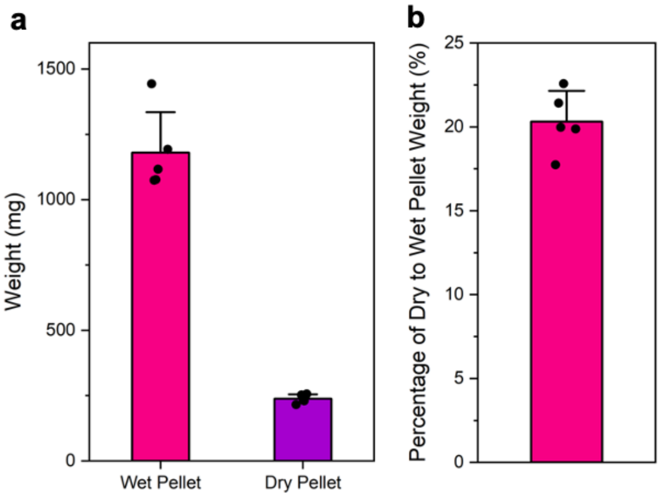

Supplementary Figure 10. Weight analysis. a Weight of wet and dry pellet of curli biofilm obtained from 500 ml cultures. **b** Percentage of dry to wet pellet weight of curli biofilm. *n* = 5. Data represented as mean ± standard deviation.

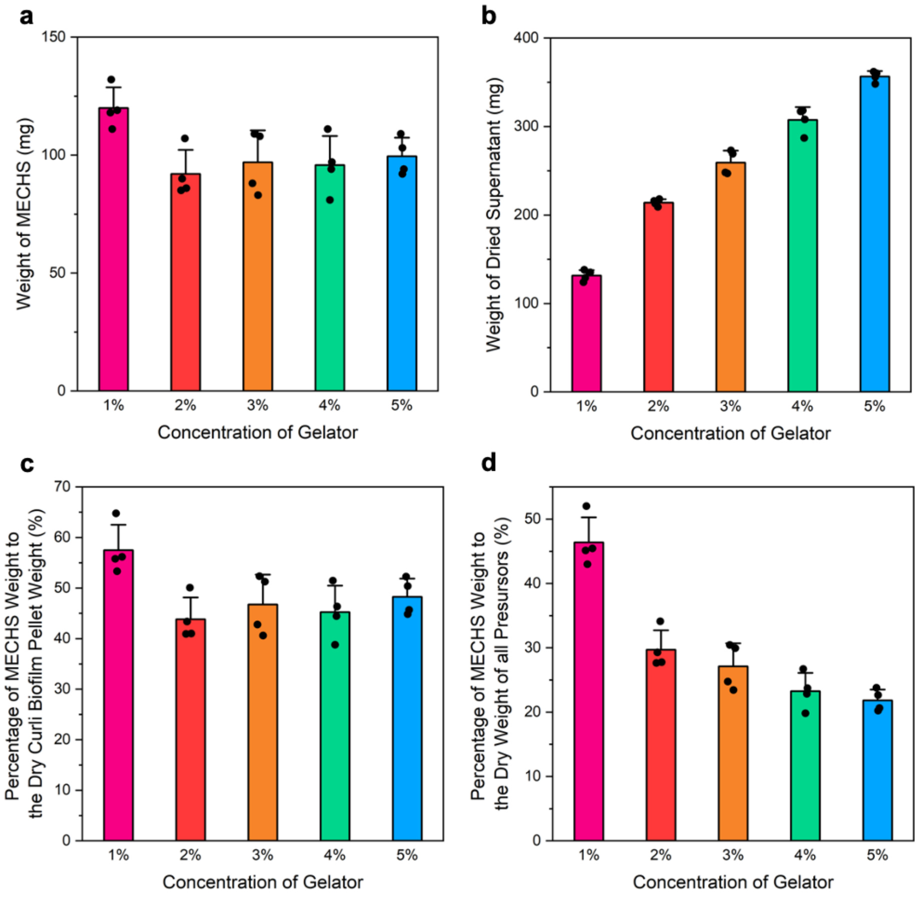

Supplementary Figure 11. Weight analysis of gelator treated MECHS. Weight of **a** MECHS films and **b** the dried supernatant obtained from 1 to 5% of gelator. Percentage of MECHS film weight to **c** dry curli biofilm pellet weight (20.3% of wet pellet) and **d** dry weight of all precursors for 1 to 5% of gelator. All precursors correspond to weights of cell pellet and that of 1-5% gelator. n = 4. Data represented as mean ± standard deviation.

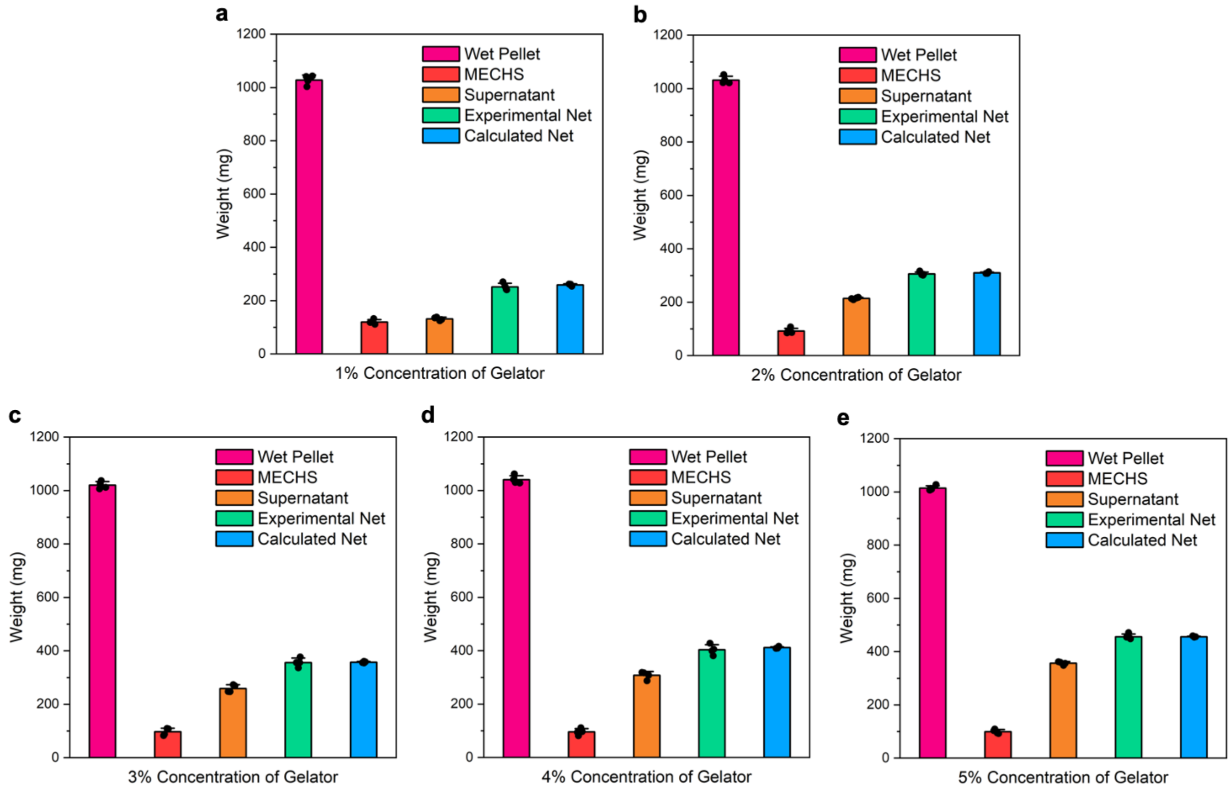

Supplementary Figure 12. Weight analysis of gelator treated MECHS. Weights of wet pellet, MECHS film, dried supernatant, experimental net (MECHS and dried Supernatant) and calculated theoretical net (20.3% of wet pellet) for **a** 1%, **b** 2%, **c** 3%, **d** 4% and **e** 5% of gelator. n = 4. Data represented as mean ± standard deviation.

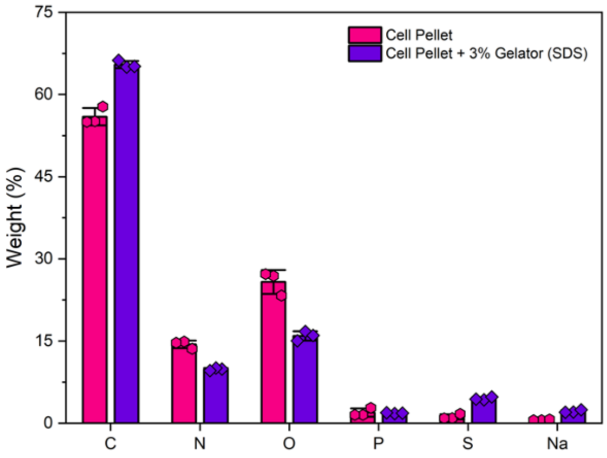

Supplementary Figure 13. Compositional analysis of MECHS. Energy Dispersive X-ray Analysis (EDAX) of *E. coli* curli biofilm cell pellets pretreated with or without 3% gelator (SDS) shows the weight percentage of various elements. n = 3. Data represented as mean ± standard deviation.

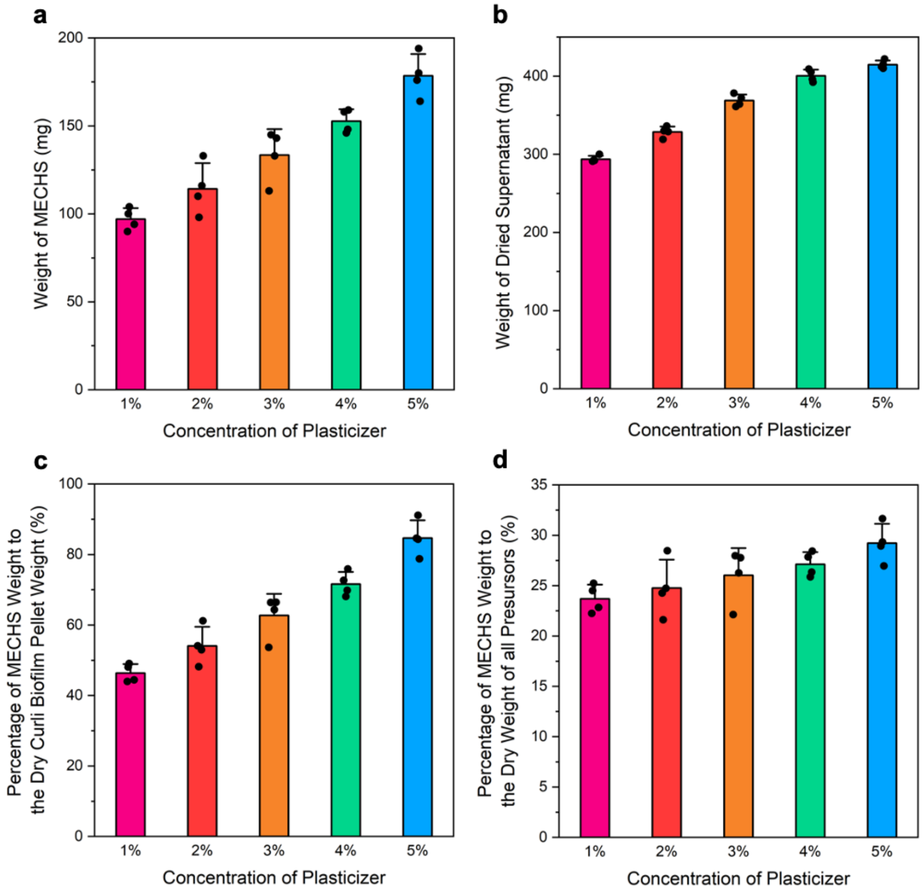

Supplementary Figure 14. Weight analysis of plasticizer treated MECHS. Weight of **a** MECHS films and **b** the dried supernatant obtained from 1 to 5% of plasticizer. Percentage of MECHS film weight to **c** dry curli biofilm pellet weight (20.3% of wet pellet) and **d** dry weight of all precursors for 1 to 5% of plasticizer. All precursors correspond to weights of cell pellet and that of 3% gelator and 1-5% plasticizer. *n* = 4. Data represented as mean ± standard deviation.

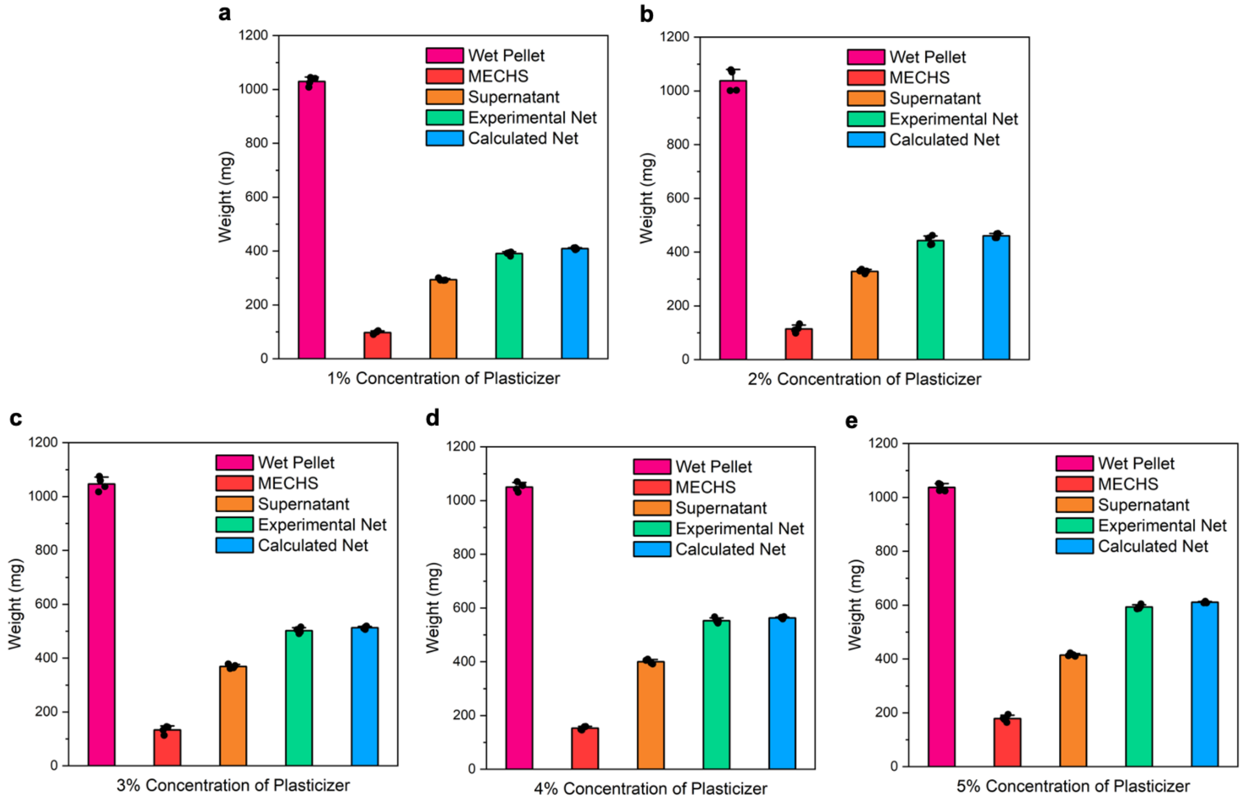

Supplementary Figure 15. Weight analysis of plasticizer treated MECHS. Weights of wet pellet, MECHS film, dried supernatant, experimental net (MECHS and dried Supernatant) and calculated theoretical net (20.3% of wet pellet) for **a** 1%, **b** 2%, **c** 3%, **d** 4% and **e** 5% of plasticizer. *n* = 4. Data represented as mean ± standard deviation.

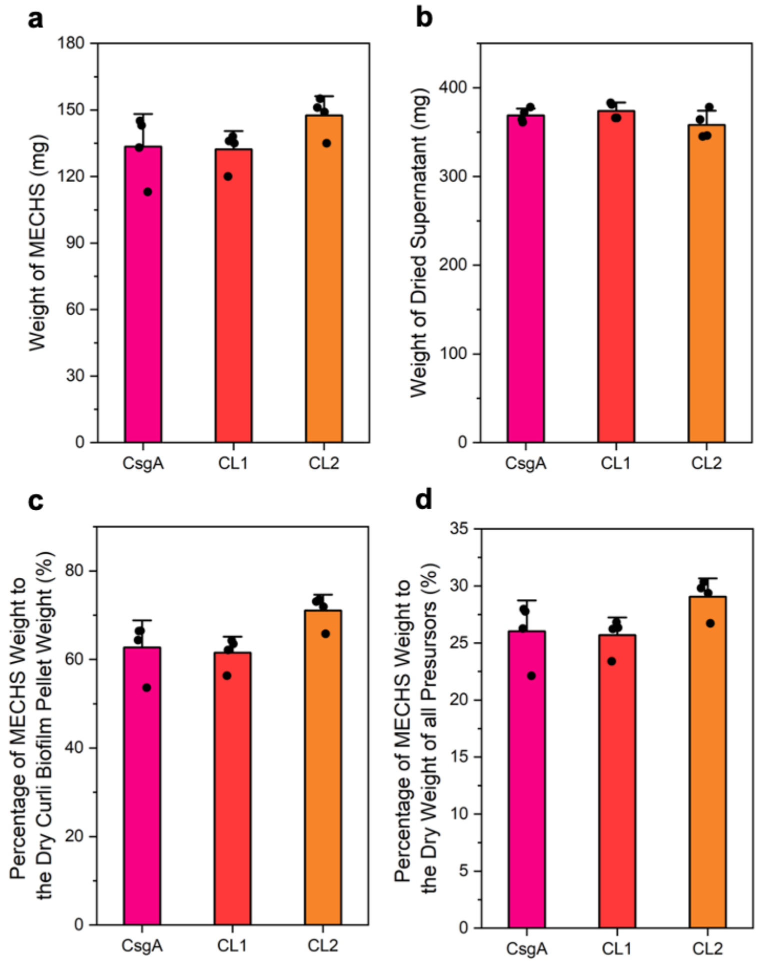

Supplementary Figure 16. Weight analysis of covalently crosslinked MECHS. Weight of **a** MECHS films and **b** the dried supernatant of CsgA, CL1 and CL2. Percentage of MECHS weight to **c** dry curli biofilm pellet weight (20.3% of wet pellet) and **d** dry weight of all precursors for CsgA, CL1 and CL2. All precursors correspond to weights of cell pellet and that of 3% gelator and 3% plasticizer. *n* = 4. Data represented as mean ± standard deviation.

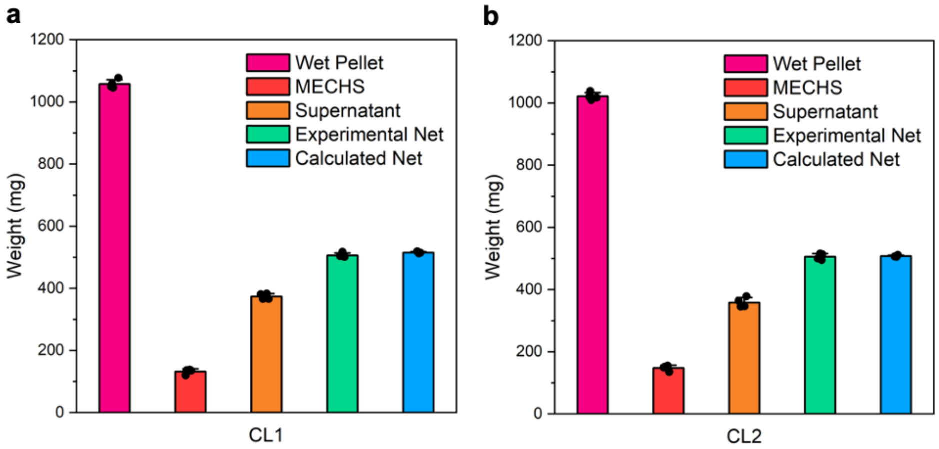

Supplementary Figure 17. Weight analysis of covalently crosslinked MECHS. Weights of wet pellet, MECHS, dried supernatant, experimental net (MECHS and dried Supernatant) and calculated theoretical net (20.3% of wet pellet) for **a** CL1 and **b** CL2. *n* = 4. Data represented as mean ± standard deviation.

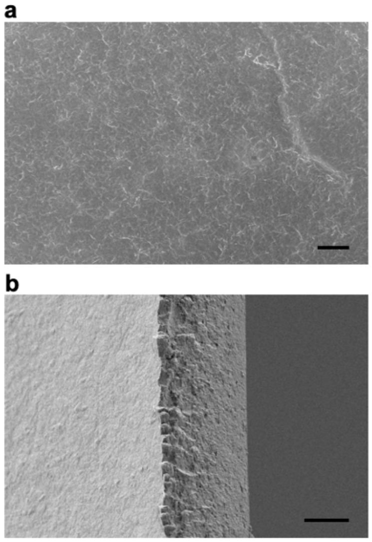

Supplementary Figure 18. Morphological analysis of MECHS. FESEM image of **a** top view and **b** side view of MECHS film obtained from 3% gelator. Scale bar **a** 20 µm and **b** 10 µm.

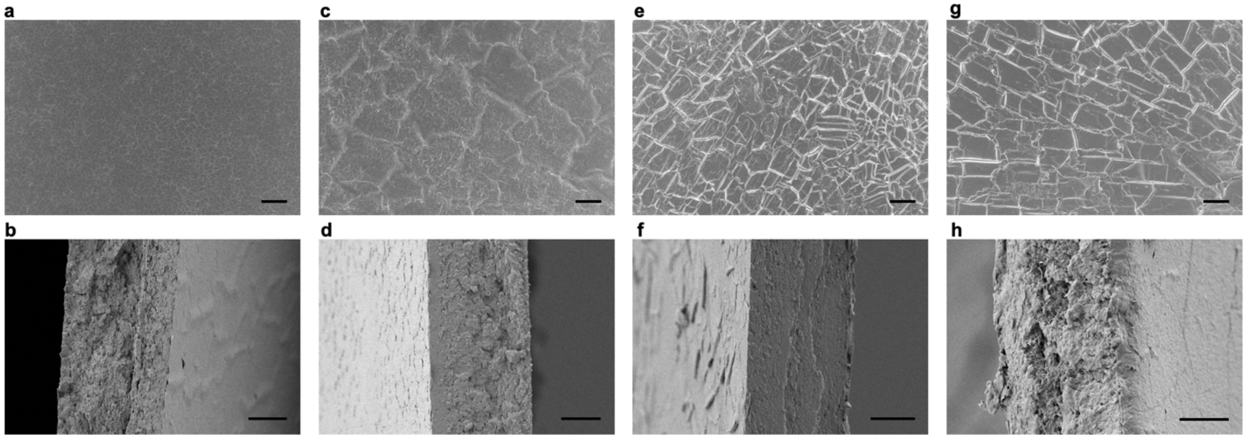

Supplementary Figure 19. Morphological analysis of MECHS. FESEM image of **a**,**c**,**e**,**g** top view and **b**,**d**,**f**,**h** side view of MECHS film obtained from **a**,**b** 1%, **c**,**d** 2%, **e**,**f** 4% and **g**,**h** 5% plasticizer. Scale bar Top Row: 20 μm and Bottom Row: 10 μm.

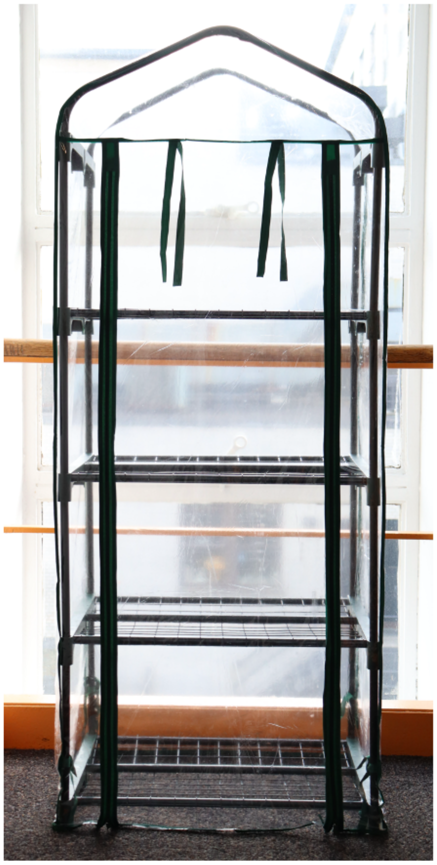

Supplementary Figure 20. Greenhouse for biodegradation experiment. Photograph of a mini greenhouse setup utilized for testing the compostability of MECHS.

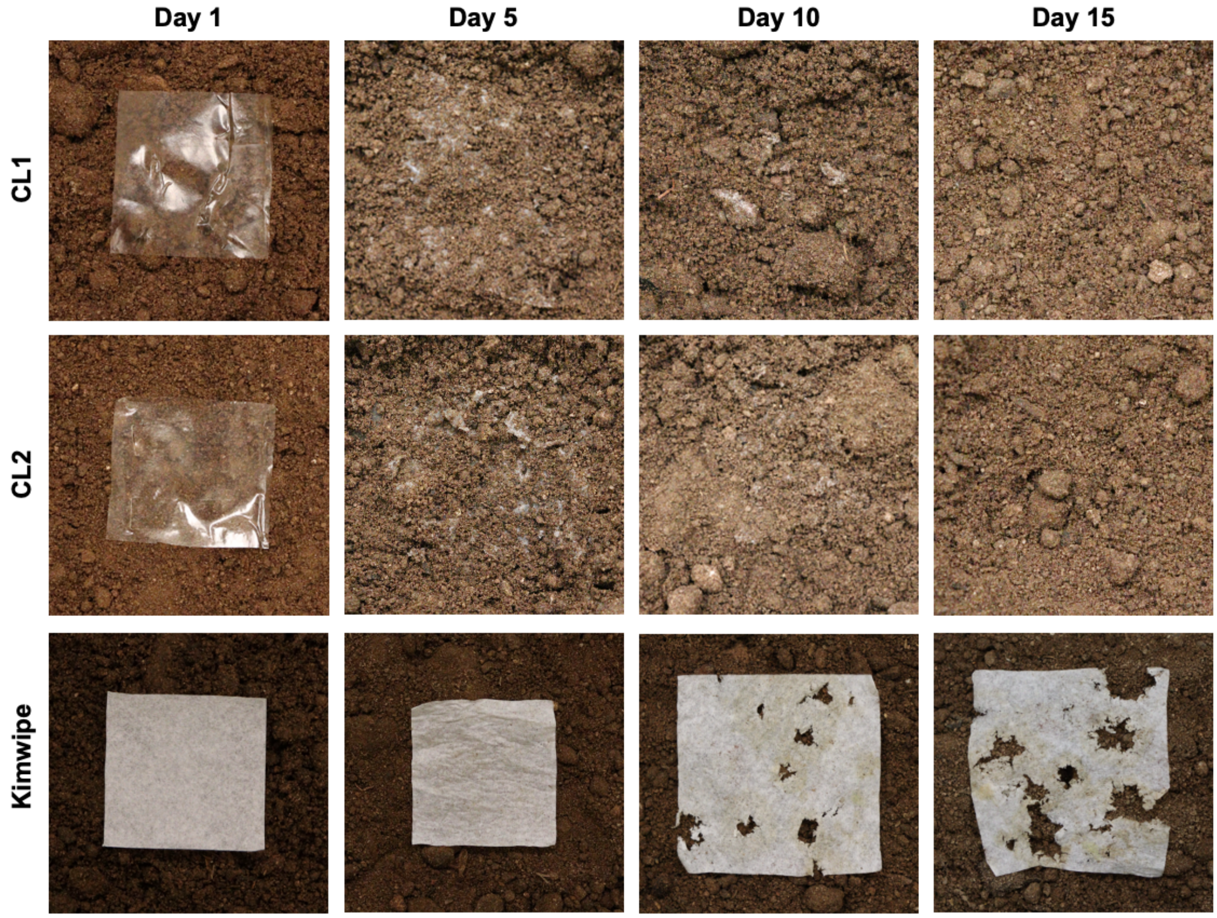

Supplementary Figure 21. Compostability of MECHS and Kimwipe in a fresh fishnure. Photographs show the biodegradation of CL1, CL2 and Kimwipe in a fresh fishnure. The lateral dimensions of the samples were 5 cm by 5 cm.

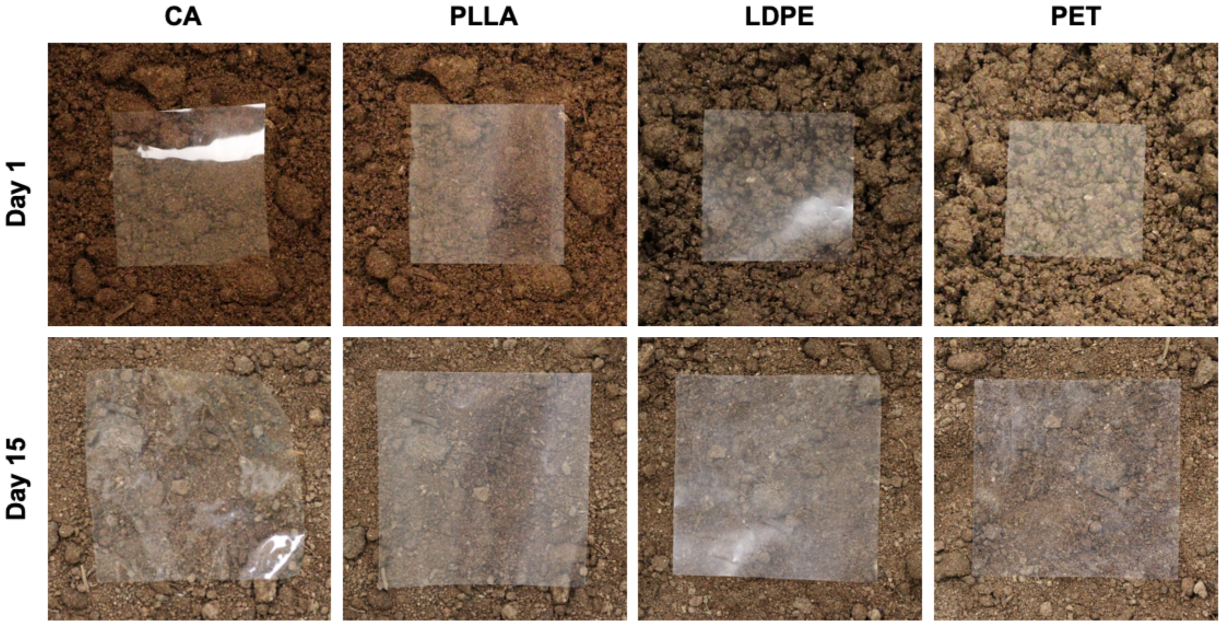

Supplementary Figure 22. Compostability of Bioplastics and Plastics in a fresh fishnure. Photographs show the biodegradation of cellulose acetate (CA), poly-L-lactic acid (PLLA), low density polyethylene (LDPE) and polyethylene terephthalate (PET) in a fresh fishnure. The lateral dimensions of the samples were 5 cm by 5 cm.

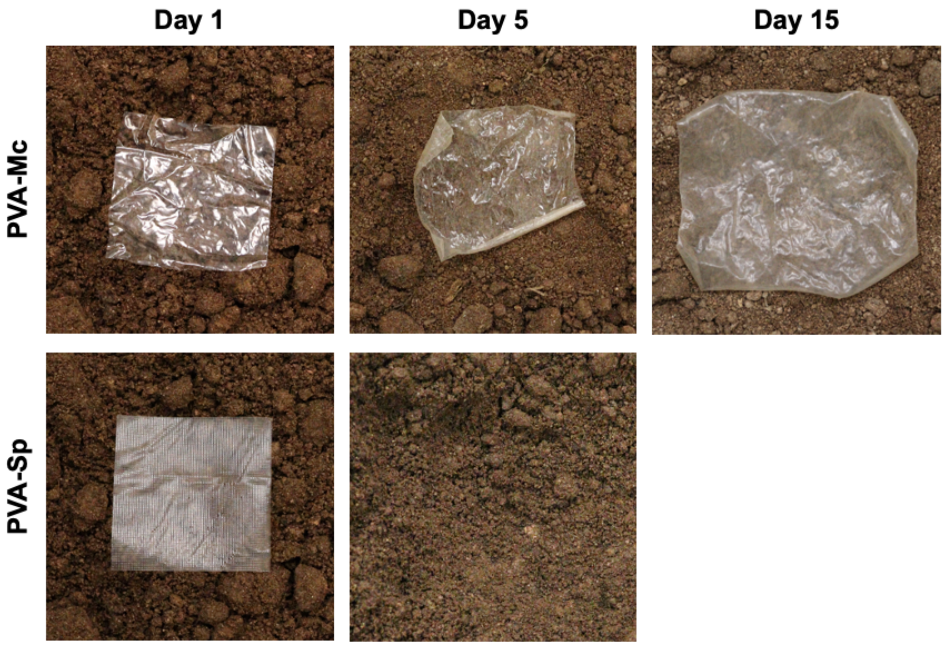

Supplementary Figure 23. Compostability of Polyvinyl alcohol in a fresh fishnure. Photographs show the films of Polyvinyl alcohol - Mckesson (PVA-Mc) and Polyvinyl alcohol - Superpunch (PVA-Sp) in a fresh fishnure. The lateral dimensions of the samples were 5 cm by 5 cm.

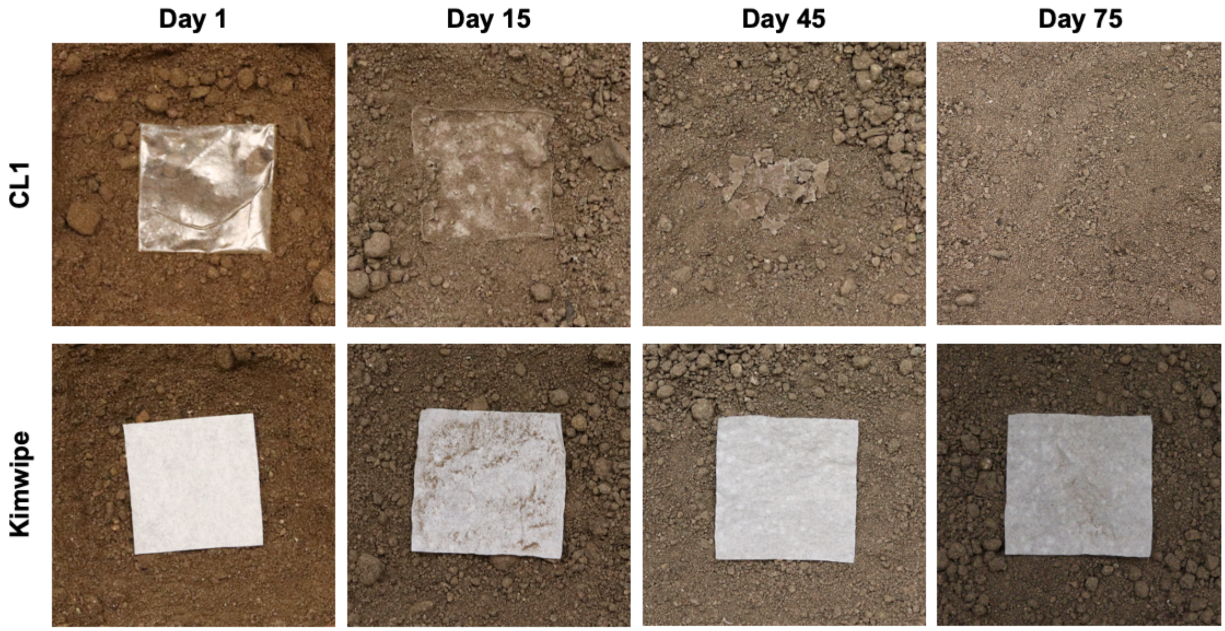

Supplementary Figure 24. Compostability of MECHS and Kimwipe in a dry fishnure. Photographs show the biodegradation of CL1 and Kimwipe in a dry fishnure. The lateral dimensions of the samples were 5 cm by 5 cm.

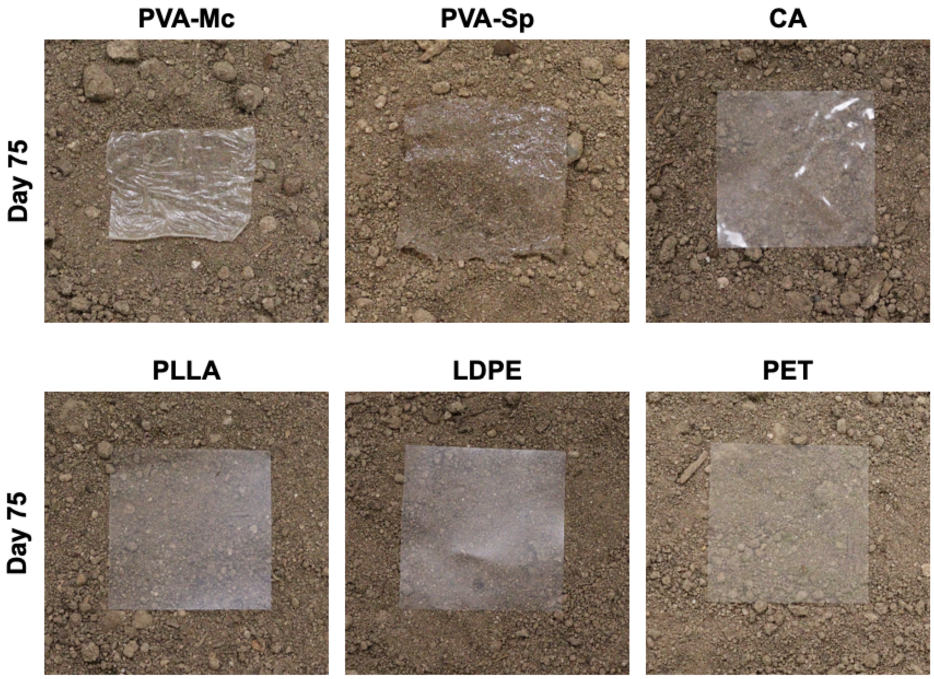

Supplementary Figure 25. Compostability of Bioplastics and Plastics in a dry fishnure. Photographs show the biodegradation of Polyvinyl alcohol - Mckesson (PVA-Mc) and Polyvinyl alcohol - Superpunch (PVA-Sp), cellulose acetate (CA), poly-L-lactic acid (PLLA), low density polyethylene (LDPE) and polyethylene terephthalate (PET) in a dry fishnure. The lateral dimensions of the samples were 5 cm by 5 cm.

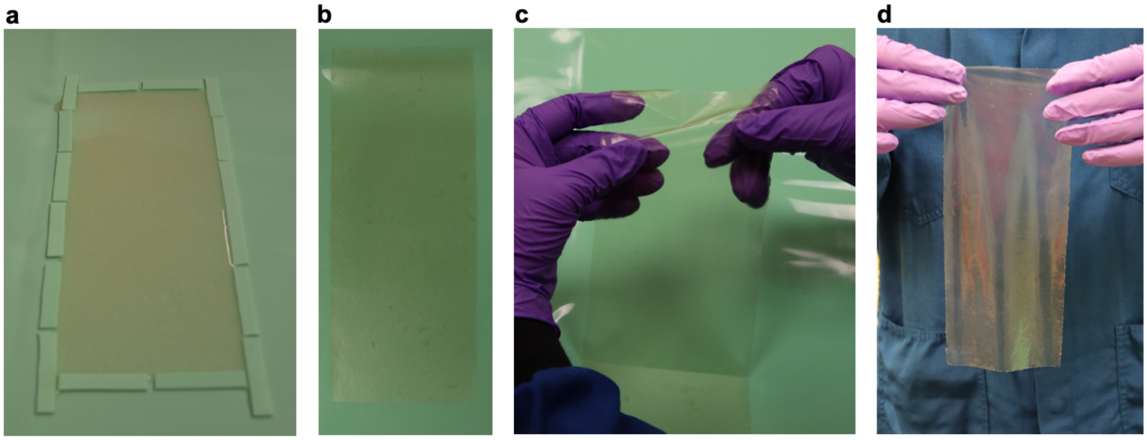

Supplementary Figure 26. Biofabrication of large MECHS prototype. Photographs show **a** casting of curli biomass on to a silicone mold, **b** ambient dried MECHS, **c** peeling of MECHS film from the silicone mold and **d** free-standing flexible MECHS film of lateral dimension 10 cm by 25 cm.

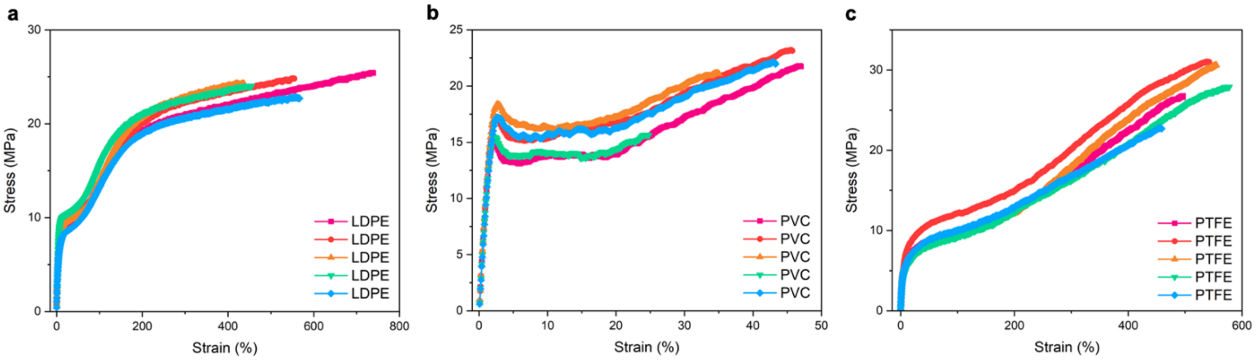

Supplementary Figure 27. Tensile tests. Stress strain curves of **a** Low density polyethylene (LDPE), **b** Polyvinyl chloride (PVC) and **c** Polytetrafluoroethylene (PTFE).

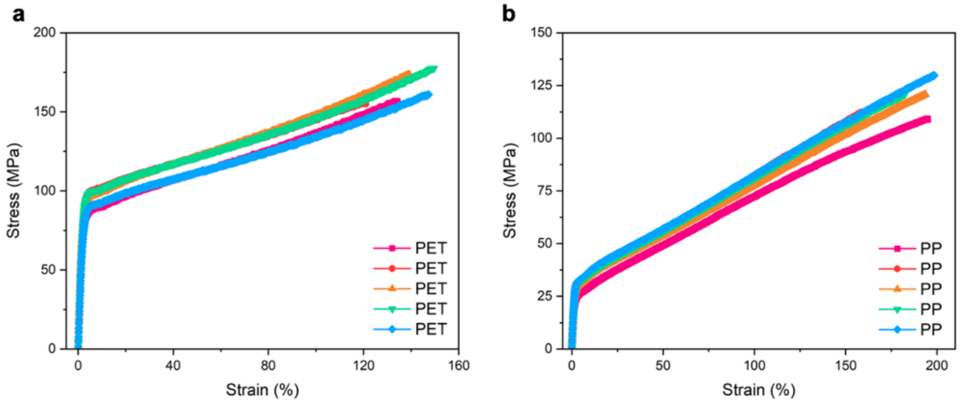

Supplementary Figure 28. Tensile tests. Stress strain curves of **a** Polyethylene terephthalate (PET) and **b** Polypropylene (PP).

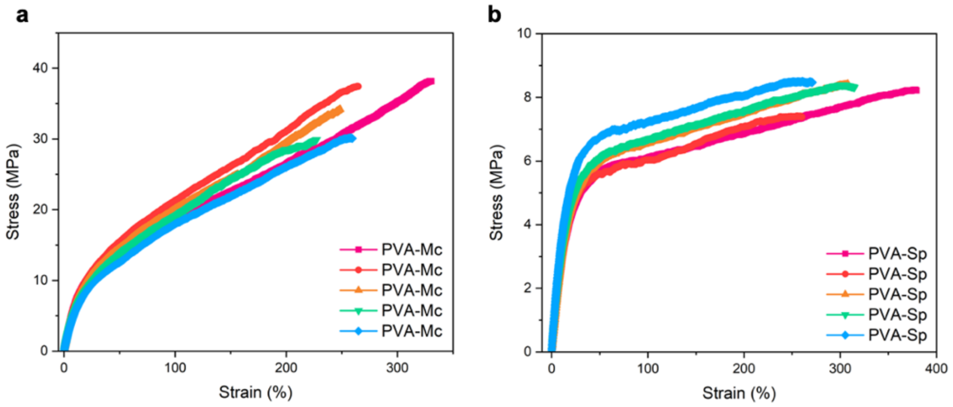

Supplementary Figure 29. Tensile tests. Stress strain curves of **a** PVA-McKesson and **b** PVA-Superpunch. PVA: Polyvinyl alcohol.

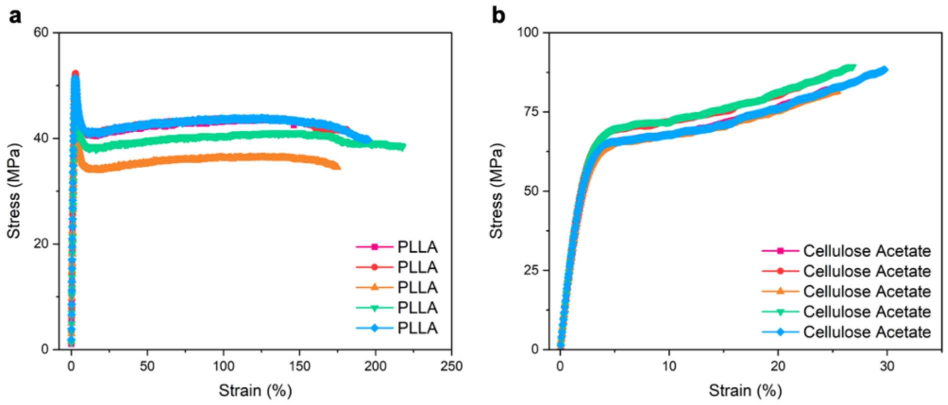

Supplementary Figure 30. Tensile tests. Stress strain curves of **a** Poly-L-lactic acid (PLLA) and **b** Cellulose Acetate.

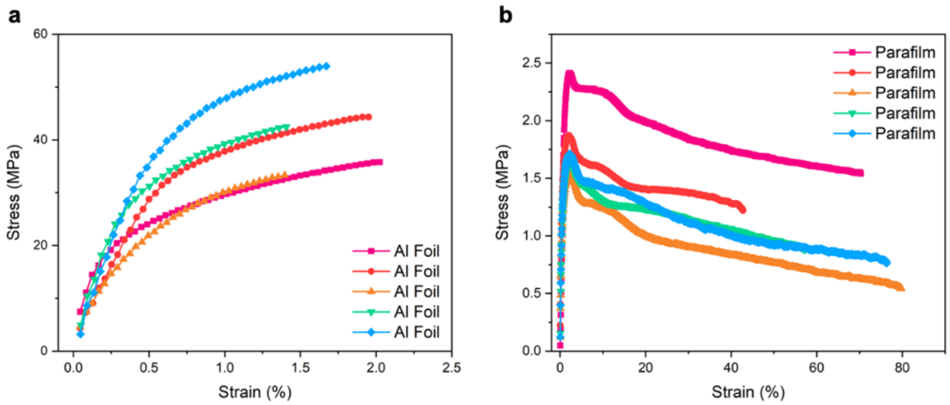

Supplementary Figure 31. Tensile tests. Stress strain curves of **a** Aluminum foil and **b** parafilm.

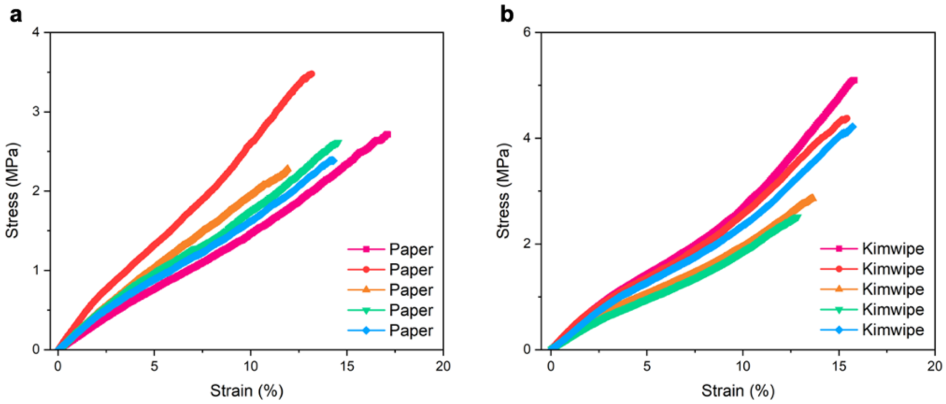

Supplementary Figure 32. Tensile tests. Stress strain curves of **a** Toilet paper and **b** Kimwipe.

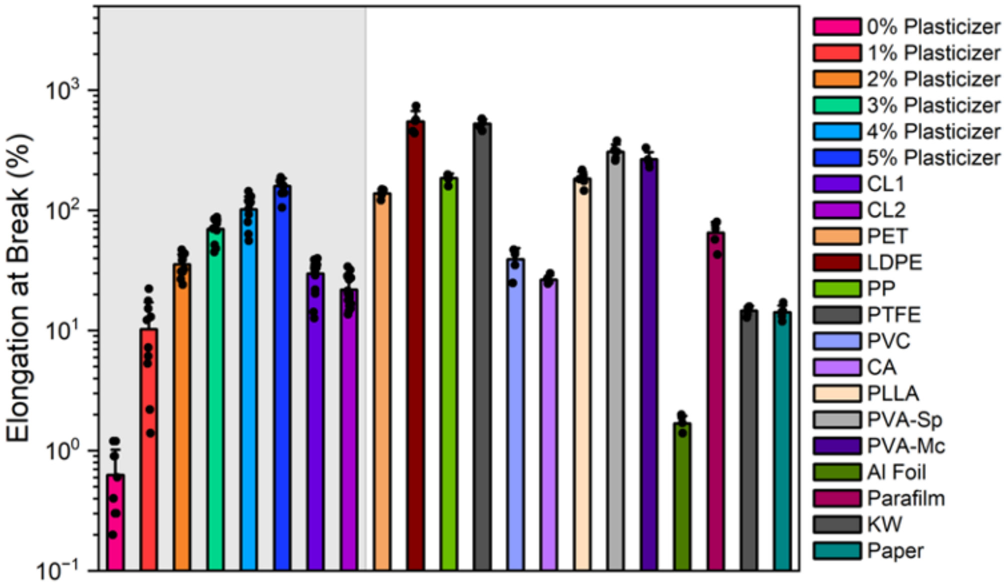

Supplementary Figure 33. Tensile tests. Plot shows the elongation at break for MECHS, various synthetic materials and biomaterials. Biological replicates *n* = 10 for 0-5% plasticizer, *n* = 15 for CL1, *n* = 20 for CL2 and *n* = 5 for all other samples. Low density polyethylene (LDPE), Polytetrafluoroethylene (PTFE), Poly-L-lactic acid (PLLA), Polyethylene terephthalate (PET), Cellulose acetate (CA), Polypropylene (PP), Polyvinyl chloride (PVC), Polyvinyl alcohol - Superpunch (PVA-Sp), Polyvinyl alcohol - Mckesson (PVA-Mc), Aluminum foil (Al Foil), Parafilm, Kimwipes (KW) and Toilet paper.

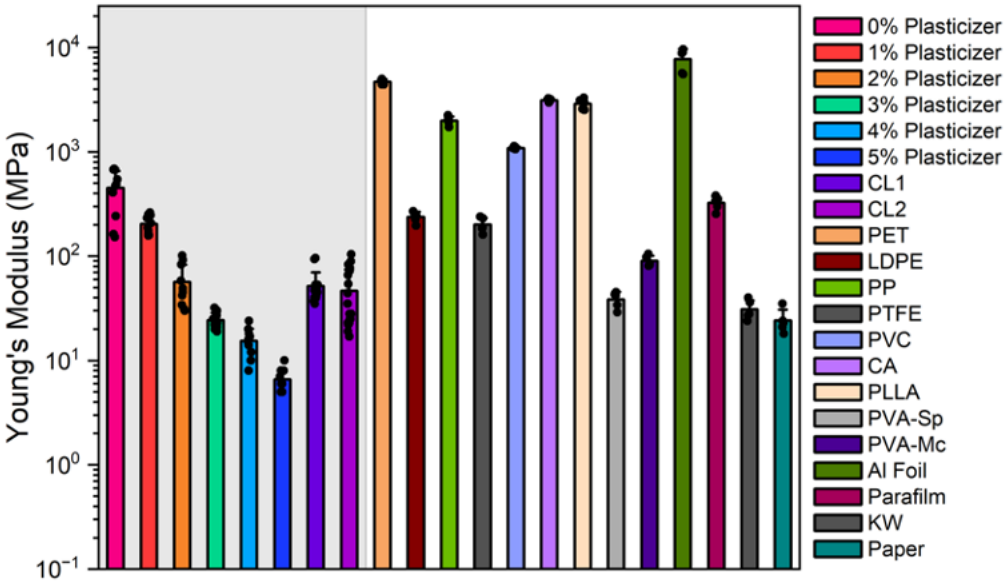

Supplementary Figure 34. Tensile tests. Plot shows the Young's modulus for MECHS, various synthetic materials and biomaterials. Biological replicates *n* = 10 for 0-5% plasticizer, *n* = 15 for CL1, *n* = 20 for CL2 and *n* = 5 for all other samples. Low density polyethylene (LDPE), Polytetrafluoroethylene (PTFE), Poly-L-lactic acid (PLLA), Polyethylene terephthalate (PET), Cellulose acetate (CA), Polypropylene (PP), Polyvinyl chloride (PVC), Polyvinyl alcohol - Superpunch (PVA-Sp), Polyvinyl alcohol - Mckesson (PVA-Mc), Aluminum foil (Al Foil), Parafilm, Kimwipes (KW) and Toilet paper.

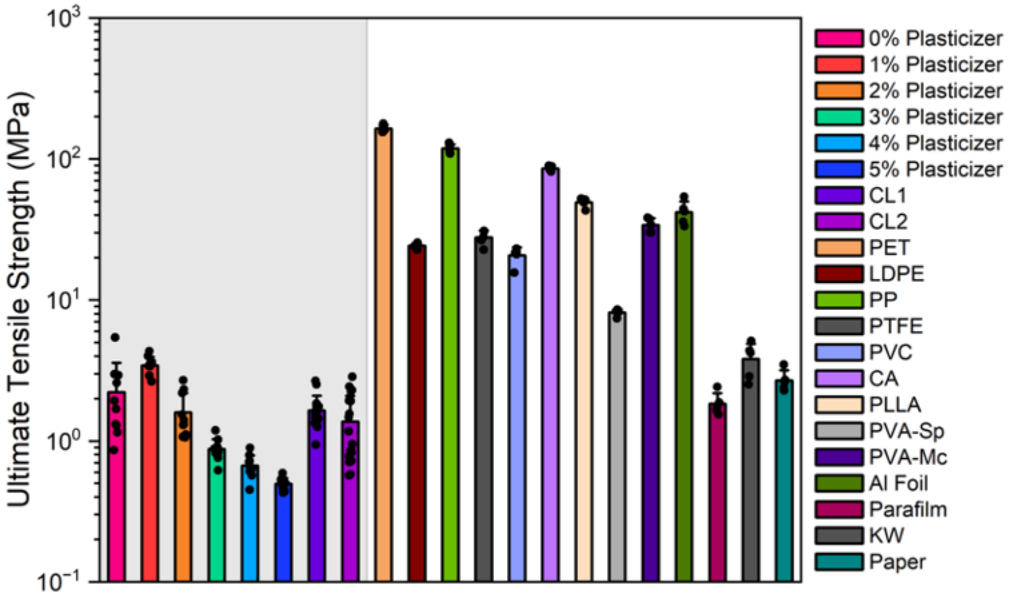

Supplementary Figure 35. Tensile tests. Plot shows the ultimate tensile strength for MECHS, various synthetic materials and biomaterials. Biological replicates n = 10 for 0-5% plasticizer, n = 15 for CL1, n = 20 for CL2 and n = 5 for all other samples. Low density polyethylene (LDPE), Polytetrafluoroethylene (PTFE), Poly-L-lactic acid (PLLA), Polyethylene terephthalate (PET), Cellulose acetate (CA), Polypropylene (PP), Polyvinyl chloride (PVC), Polyvinyl alcohol - Superpunch (PVA-Sp), Polyvinyl alcohol - Mckesson (PVA-Mc), Aluminum foil (Al Foil), Parafilm, Kimwipes (KW) and Toilet paper.
